# What GPs mean by 'spirituality' and how they apply this concept with patients: a qualitative study

**DOI:** 10.3399/bjgpopen18X101469

**Published:** 2018-04-18

**Authors:** Alistair Appleby, John Swinton, Philip Wilson

**Affiliations:** 1 GP, Aviemore Medical Practice, Aviemore, UK; 2 Chair of Divinity and Religious Studies, School of Divinity, History and Philosophy, King's College, University of Aberdeen, Aberdeen, UK; 3 Director, Centre for Rural Health, University of Aberdeen, Centre for Health Science, Inverness, UK

**Keywords:** primary care, general practice, spirituality

## Abstract

**Background:**

Little is known about how the concept of spirituality is understood and applied by GPs.

**Aim:**

To provide a detailed description of how GPs understand the concept of spirituality and apply this with patients.

**Design & setting:**

Nineteen Scottish GPs were interviewed about their perceptions of the concept of spirituality and how they apply this in practice.

**Method:**

An approach informed by grounded theory was used to identify and summarise common themes.

**Results:**

Seven concepts concerning spirituality emerged, some of which are previously unrecognised. Four attitudes to spiritual care and four patterns of spiritual care were identified.

**Conclusion:**

GPs have varying views on what spirituality is, and these relate partly to individual beliefs and experiences. These create considerable variation in the delivery of spiritual care.

## How this fits in

Some GPs understand themselves to have a role in spiritual care, chiefly in the context of palliative situations. A much wider range of opinion and practice regarding spirituality exists than was previously recorded. These variations are likely to relate partly to GP’s philosophical frameworks and assumptions. Further conceptual work may be required if general practice is expected to deliver spiritual care through teams with diverse frameworks of belief. Variations in spiritual care approach need to be further evaluated for patient acceptability and outcomes.

## Introduction

There is little published evidence of basic groundwork which ascertains how GPs perceive the concept of spirituality, or apply this in practice. This study aims to fill this gap.

The small number of studies that have already been conducted in Europe largely tend to provide or assume a concept of spirituality.^1–3^ Only one US-based study has explicitly looked at how GPs themselves define and understand spirituality.^4^ While limited guidance currently exists for GPs on spiritual care,^5^ its focus is on the provision of chaplaincy services,^6^ and speaks to limited contexts such as palliative care and mental health.^7,8^

Previous research suggests most patients are interested in spiritual discussion,^9^ but few studies have attempted to examine exactly what GPs do when they talk about delivering spiritual care, and some of their claims may simply constitute good active listening.^10^ While it is clear that GPs sometimes refer to chaplaincy services in palliative care contexts, and that at least one spiritual assessment tool has been assessed for usefulness with GPs,^3^ the present authors postulate that a much wider range of spiritual care approaches might be being provided.

### Cultural context

Most European researchers consider spirituality to be different from, but related to, religious belief, and it is now becoming clear that it cannot be assumed that simple measures like church attendance^11^ are reliable indications of a population’s spiritual affiliation or interest, or even personal religious belief or practice.^12,13^ In Europe, a much more complicated picture now exists, with non-religious forms of spirituality, minority faiths, and disaffiliated religious belief gaining greater significance.^14,15^

## Method

### Study population and sampling

Studies in spirituality must decide whether they seek a polyreligious diversity — where participants from many religious groups are sampled — or a sample diverse in its affinity to a wider, and not necessarily religious, concept of spirituality. The latter, which accepts a spiritual affinity may vary in intensity and may be expressed religiously or not, is similar to that developed by the World Health Organization,^16^ and that contained in key NHS resources and consensus statements.^17,18 ^This study aimed to sample this wider type of diversity, and therefore religious or denominational categorisations are of less importance. Asking for religious orientation may result in participants giving institutional answers rather than personal ones, or produce a feeling of being judged by the interviewer or process.

A purposive sample of GPs, based on a sampling strategy, was obtained using email, mailout, word of mouth, and 'snowballing' strategies. The sampling strategy identified GPs with a mix of sex, experience, and strength of spiritual affiliation. The views of GPs in northern Scotland were initially sought. Using participants as informants, the sample was later broadened to other areas of Scotland, where it was identified that alternative viewpoints were present. Recruitment continued until saturation, where the research team was satisfied that the major themes showed depth and variation.^19^

### Interviewing

Nineteen GPs were interviewed by a male GP with qualitative interview training. Interviews were conducted at a place of the interviewee's choosing, usually the workplace, and lasted 40–65 minutes. Themes included exploring what GPs understood by the term spirituality, their attitudes to providing spiritual care, and their experiences in applying the concept to care. The interviewer sought to mirror the language of the interviewee and did not offer definitions. Interviews were transcribed verbatim and anonymised prior to distribution to the research team.

### Coding and analysis: interpretation of data

The interviews were examined line by line for common themes. Coding and analysis was performed using the constant comparative method described by Merriam,^20^ and was informed by grounded theory.^21^ A mixture of a priori and emerging codes were applied. The team discussed emerging themes, and their relationships and significance. During this interpretive phase, reference was also made to critical realist theory in order to avoid disengaging the GPs' stories from their context, and to avoid purely reductionist or constructivist interpretations.^22,23^

## Results

Nineteen GP interviews were conducted. The age range was 36–60 years, and experience ranged from 3–31 years in practice. Twelve were male and seven female. Ten were GP trainers and nine were not. Most of the GPs were partners, but some were locums or had portfolio jobs.

### GPs’ personal spiritual orientation

Despite the lack of formal questioning, some GPs made explicit statements about their own spiritual, religious, or philosophical orientation. These statements included differing levels of commitment to theism, agnosticism, atheism, and 'human need' spiritual frameworks.

### Concepts of spirituality

Seven themes concerning the concept or definition of spirituality emerged from the interviews.

#### Spirituality is a meaningless concept

These GPs considered the word 'spiritual' to be a non-entity, to be better expressed in other terms, or to be an invalid construction. There was a tendency to connote spirituality closely with religion in this group. They used the word 'religion' more than other groups, and tended to be personally non-religious or atheist:

*'Um, I ... despite having thought about it quite a lot, I’m still not convinced that spirituality’s a valid concept. Um, I hear the words, the word used a lot, particularly in relation to, for example, palliative care, but I actually don’t know what it’s on about.'* (Participant 8)

#### Spirituality as an unclear concept

Some GPs expressed uncertainty or confusion about the concept or definition of spirituality, and about whether there is an agreed definition, but conceded that it might have individual relevance to a patient:

*'I think I’d find it hard to say in an easy sentence what I, what I believe, um, spirituality* [is] in *practice.'* (Participant 1)

#### Spirituality as a useful concept

Some GPs expressed spirituality as a useful concept, which would have clinical utility on a pragmatic basis. 

#### Spirituality as a psychological need

Others described spirituality as a psychological need, centred around a human cognitive or emotional need.

#### Spirituality as personal meaning

Some GPs used a concept of spirituality based on spirituality as personal meaning; the deep meaning and values an individual patient holds about their own life.

#### Spirituality as integral to humanity

Other themes spoke of spirituality as integral to humanity; as a deep and possibly fundamental aspect of personhood, or a shared aspect of all humanity:

*'I think I probably have a slightly looser understanding of it now as being more about somebody’s, um, sort of belief set and what matters to them, more from the meaning of life point of view, and that needn’t necessarily be about a defined religion, be it Christian or Muslim; um, for some people it’ll have a looser, less rigid feeling but really what makes some, somebody, somebody’s belief set about the greater meaning of life and things that are important to them.'* (Participant 3)

#### Spirituality as divine connection or relationship

This theme suggested spirituality as referencing a divine being, spiritual realm, or spiritual reality which has independent existence. Most of the GPs who expressed this view had religious affiliation. Some did not, but espoused values of personal spiritual awareness or quest:

*'If you’re trying to give a formal definition, a conventional Christian view would be that your body, soul, and spirit, your physical shell, your emotional being, and that part of you which is in touch with God, as you see him. For those that don’t, there’s still that element of the greater spiritual awareness in the world and the universe that people have intrinsically as part of their being.'* (Participant 19)

These views were not mutually exclusive; for example, GPs who expressed divine connection views concerning the concept of spirituality frequently also held views of spirituality as a shared or basic human need. Some GPs’ views developed as they thought through the issue, in one case moving from 'uncertainty' to 'deep human need' constructs through internal dialogue (Participant 7).

### Attitudes to providing spiritual care

Four distinct attitudes to providing spiritual care were present in the GPs’ narratives.

#### Rejecting

These GPs expressed an unwillingness to be involved in spiritual care. Reasons included: concerns that a spiritual approach may not help, or is invalid; the possibility of disrupting the relationship with the patient; or the possibility of overstepping a role boundary. There were often personal philosophical reasons. One GP spoke of his discomfort when patients revealed 'deluded' religious beliefs. Another ventured:

*'I find the question quite difficult to answer, from a personal point of view, because I don’t really accept that there is a concept of spirituality, so … given that, it’s difficult to see how … it’s difficult to see how it applies to primary care.'* (Participant 8)

#### Guarded or provisional

These GPs had concerns about GP involvement in spiritual care and wished to be involved only to a minor degree or in certain restricted circumstances:

*'I might say, "Well actually your spirituality, very important to health outcomes, you need somebody to look at your spiritual health." Am I the right person to be doing that or would that, would there be somebody else that might be more skilled that we could work with? I might ask the question, and then signpost you, but is it right for me to be dealing with that?'* (Participant 3)

#### Pragmatic

These participants often took an 'if it helps' type of view. They saw a role for themselves in spiritual care and were open to its provision, though often put limits on that. They expressed ideas around encouraging patients to find meaning and purpose. They were not always clear about who would provide spiritual care and sometimes expressed reservations about the practicalities of this:

*'I think spirituality has a place. I’m not sure I’m the person to deliver that, in my role as a GP. Erm, I certainly feel there is room for spirituality in general practice, erm, I’m not sure who would deliver that. It may be a list of potential sources, be it your local vicar down to your, you know, your reiki healer or, you know, there may be a resource there for patients to access.'* (Participant 4)

#### Embracing

This group expressed willingness or enthusiasm for a role in spiritual care. They often related this to a belief that spirituality is a fundamental aspect of humanity, in some way foundational to health, or as an opportunity to bring an alternative resource or approach to their patients. Some related this to a sense that GPs were skilled at addressing spiritual issues. Others related this to their own spiritual or religious belief:

*'I think so many of us reach for the prescription pad and prescribe when we ought to just be slowing down and looking at these deepest inner needs or spiritual needs, and accepting that we are the new, in some ways, the new clergy, the new priests that people come to, and embracing that because we’re good at it, but going that wee bit further into the spiritual issues.'* (Participant 17)

### Spiritual care patterns

When faced with what the participants saw as spiritual issues, participants described four distinct modes of action. These were not clearly circumscribed but followed patterns which had certain characteristics. A majority of GPs offered more than one pattern of spiritual care, though some had strong preferences for one.

#### Invalidating

Some participants indicated they were very unlikely to venture any exploration of spirituality or make any attempt at spiritual or religious care, even in a palliative care context. These GPs indicated that they, as GPs, steered the discussion back to other areas of care if spirituality or religion was mentioned by the patient. One GP (Participant 16) alluded to returning the conversation to 'real things' and to personal discomfort in discussing patients’ spiritual lives or religion:

*'It’s never really come up. I mean, we have regular palliative care meetings, but a Macmillan Nurse comes and we discuss the patients, and she has certainly discussed these matters with the patients, but she’s never felt that it was important enough to raise about, er, anyone in particular. So I’m not sure, I don’t perceive it as a real issue or real problem, no.'* (Participant 16)

Another GP felt it was not their job to fix spiritual crises:

*'Um, so I think the answer is I don’t, I don’t go there. I don’t really understand. I don’t really understand the concept. I can, you know, intellectually I can get the fact that some people, um, have spiritual crises, as you know, they might be called, whereby they are doubting their beliefs, or are some way feeling that they’ve failed their God or whatever and that’s a bit of a pity. But I ... there’s probably not much I can do about that.'* (Participant 8)

GPs who described this pattern of spiritual care described themselves in non-religious or atheist terms. These GPs showed evidence of also delivering cultural-instrumental care (collecting information about religious customs of relevance to practical health decisions). Not all GPs who were personally non-religious or atheist expressed invalidating care patterns.

#### Cultural-instrumental

Several participants indicated that they used an understanding of patients’ beliefs, particularly their religion, as cultural information or clinically relevant background:

*'I think that it’s important to know what religion, whether, whether somebody who’s dying has a religion and it’s important to know ... what cultural background people are from, in terms of the way we practice medicine, in some respects, um, when you’re dealing with people in particular from minority communities so, um, I, I think it’s, it’s, it’s of some practical value to know that people are Muslim or that people are Jewish.'* (Participant 8)

This information was viewed as having practical utility; that is, helping the GP to understand the patient’s personal views or preferences, or how the GP may be expected to act. These accounts imply that the GP is external to the patient’s spiritual reality, observing and sometimes respecting it, without engaging with it as a shared experience or common reality. Respect was spoken of in terms of entitlement to a religious view which might be different from or even incompatible with the GP’s own view.

These interviews contain clues that the GP concerned felt that their own and the patient’s views could neither be reconciled nor explicitly questioned in the clinical context. One GP (Participant 11) expressed discomfort at finding what he saw as fundamentalist sacred texts in a patient’s house, but felt he had to respect the patient's right to these beliefs unless they were obviously harmful to the patient or others.

#### Referring

A third spiritual care pattern emerged in which the GP would refer or direct a patient, in certain circumstances, to a spiritual advisor, chaplain, or member of the patient’s own faith community, while the GP did not see themselves as a spiritual carer or resource. These narratives tended to indicate that the patient made the GP aware of the importance of a spiritual or religious need, and the GP then flagged resources:

*'I don’t see myself as being a person with the spiritual answers but I often detect it with them and push, push them along that road if they want to.'* (Participant 5)

Some GPs felt that referring and recommending had different responsibilities in terms of duty of care; namely, a referral required certain qualities to be known about the spiritual advisor, which were not necessary for a suggestion that a spiritual advisor might be sought. Concern was sometimes expressed about the quality of spiritual care available to patients in non-mainstream faith groups. A referring pattern of spiritual care was present in narratives from participants of a wide range of personal spiritual or religious affiliation.

#### Active

The fourth pattern reported suggested active spiritual or religious dialogue with the patient; that is, a more detailed investigation of patient’s beliefs and how they affected that patient. These occurred in the contexts of palliative care, addiction, sexual abuse, and health decisions. This included clarification of the patient’s own position or beliefs, and on occasions, exploring the patient’s concept of transcendence, or relationship with the divine:

*'Spirituality for me, I think, means what are my, what are the patients’ deepest inner needs and I guess I’ve kind of based that round Maslow’s hierarchy of needs, so significance, security, self-esteem, um, and I guess moving on from there to um, a concept of transcendence from that, um, and that’s how I would try and help patients understand it, and that they need to be connected to something in these three areas of their life bigger than themselves, beyond themselves.'* (Participant 17)

In certain restricted circumstances, this led to a theological or philosophical conversation with the patient:

*' ... if people are in the last weeks and days of life that they’re needing all the comfort they can get and it is critical that, when they wish to explore the existential aspects, that I introduce it and, and maintain it as a, a, a, a, a comfort ... I want them to have every sense of support through that, so it is important that I, I would tap into their belief system, such as it is, and reinforce whatever beliefs they have regarding an almighty, eternal being, and that that is a being who seeks their best interest and will be a source of help through this horrible, literally once-in-a-lifetime experience. Um, but all the time trying to keep it within the context of me as their physician, seeking their wellbeing, not me as a proselytiser for my faith system.'* (Participant 12)

A small number of GPs reported offering to pray with or for the patient. A previous knowledge of the patient and the establishment of a degree of congruent belief seemed to precede, and possibly be a prerequisite to, these offers. These offers were typically made in difficult circumstances where conventional medical options had been exhausted:

*'I suppose it tends to be someone who I’ve known as a doctor over a number of years. We’ve talked about faith and they’ve come in a crisis and at the end I’ve done the biomedical model, but we’ve then moved on to talk about spirituality and, as I’m summing up, I’ll ask them, “Would it be good to pray now?” and they’ll say, “Yeah, that’d be fantastic”, so …'* (Participant 17)

The transcripts indicated these offers were usually welcome, but one GP (Participant 8) recounted an instance of a patient being offended by a hospital clinician’s offer of prayer.

## Discussion

### Summary

The GPs in the sample hold diverse concepts of spirituality, some having no concept at all, and the spiritual care they offer to patients varies widely. Comparative analysis suggests there is a likely association between themes of spirituality as meaningless, a rejecting attitude to spiritual care, and an invalidating spiritual care pattern. Conversely, there is a likely association between a 'divine connection' understanding of spirituality, an embracing attitude to spirituality care, and an active pattern of spiritual care.

Despite this, most GPs offered several patterns of care, and high levels of pragmatism and patient-centeredness were noted which allowed GPs, to varying degrees, to move away from their own understanding of spirituality, and encourage the patient’s own spiritual resources or frameworks as a source of help.

### Strengths and limitations

It is recognised that spiritual and religious affiliation has many dimensions, and is difficult to categorise and measure.^24^ Like all qualitative studies, this is a representation of the study participants, and further studies are needed if the conclusions are to be generalisable to other populations. It is possible that faith groups and sceptical opinions may be harder to access and therefore underrepresented. The team was multidisciplinary, including academics from the fields of theology and clinical general practice.

### Comparison with existing literature

Although some studies have recognised the difficulties with defining spirituality,^25^ previous studies have largely used a priori definitions^1–3,10,26^ or implicit definitions or concepts^27,28^ rather than investigating those which GPs understand or use. These definitions, which include consensus definitions, have generally been formed in the context of palliative care and, occasionally, researchers' and participants' definitions may seem to vary significantly.^29^

Most previous studies have been in a palliative care context. In contrast, this study allowed participants to discuss spirituality in the context in which they felt it was important. This included not just palliative care, but addiction and mental health, spiritual crisis, self-image, guilt and forgiveness, relationships, and sexual abuse.

This study provides a more in-depth description of the concept of spirituality as GPs themselves use it, including a much clearer recognition that some GPs see spirituality as meaningless, while others consider there to be importance in divine connection. This study offers a categorisation of these concepts.

Some previous studies have hinted that personal belief structures influence GPs’ attitudes to spiritual care.^29^ This study strengthens and confirms this connection. This study is the first European-based study to describe 'active' forms of spiritual care. These patterns of care included dealing with existential distress in dying patients by active engagement in theological dialogue and prayer for patients. This usually, but not always, occurred in the context of a shared religious framework.

Few previous studies have been conducted with explicit input from academics in the fields of philosophy or theology and clinical general practice.

### Implications for research and practice

Scottish GPs hold heterogeneous concepts of spirituality and offer a much wider range of approaches to spiritual care than has previously been recognised. This study demonstrates more clearly that these are likely to be related to personal attributes and previous experiences, including GPs’ own philosophical or religious views.

Research is needed into whether GPs’ views of spirituality and patterns of care are fixed or fluid, and whether they are moderated by training. Further research is needed to examine patient acceptability and outcomes related to patterns of GP spiritual care, and in multiethnic contexts.

This research suggests that several factors currently affect the willingness and ability of primary care to deliver spiritual care confidently. Training for GPs that encourages increased awareness of GPs’ own philosophical views, and how these impact care and more robust referral systems, may help. A more congruent concept of spirituality — preferably referenced to a contemporary philosophy of science, such as basic critical realism^30^ — may also enable this. Alternatively, primary care may need to accept that GPs offer differing patterns of spiritual care and more carefully define what the minimum standard of competency is, and what constitutes good care.
